# Coupling CRISPR/Cas9 and Lambda Red Recombineering System for Genome Editing of *Salmonella* Gallinarum and the Effect of *ssaU* Knock-Out Mutant on the Virulence of Bacteria

**DOI:** 10.3390/biomedicines10123028

**Published:** 2022-11-24

**Authors:** Hamza Tahir, Abdul Basit, Hafsa Tariq, Zulquernain Haider, Asim Ullah, Zafar Hayat, Shafiq Ur Rehman

**Affiliations:** 1 Institute of Microbiology and Molecular Genetics, University of the Punjab, Lahore 54590, Pakistan; 2 School of Biology, University of St Andrews, St Andrews KY16 9AJ, UK; 3 Division of Infection and Immunity, The Roslin Institute, University of Edinbrugh, Edinburgh EH8 9YL, UK; 4 Department of Animal Nutrition, University of Veterinary and Animal Sciences, Lahore 54000, Pakistan; 5 Department of Animal Sciences, University of Sargodha, Sargodha 40100, Pakistan

**Keywords:** CRISPR/Cas9, lambda recombineering, genome editing, type three secretion system, virulent genes, *Salmonella* Gallinarum, poultry experimental model

## Abstract

The poultry industry in developing countries still faces a significant threat from fowl typhoid, a disease caused by *Salmonella* Gallinarum that has been well contained in more economically developed countries. In addition to the virulence exhibited by large virulence plasmid (85 kb), Salmonella Pathogenicity Island 2 in *S*. Gallinarum plays a key role in mediating disease through its type III secretion systems (TTSS). The TTSS secrete effector protein across the *Salmonella* containing vacuoles and mediate the internalization of bacteria by modulating vesicular passage. In this study, candidate virulent *ssaU* gene (~1 kb) encoding type III secretion system was successfully deleted from indigenously isolated *S*. Gallinarum genome through homology-directed repair using CRISPR/Cas9 and lambda recombination systems. CRISPR/Cas9-based genome editing of poultry-derived *Salmonella* Gallinarum has not been previously reported, which might be linked to a lack of efficiency in its genetic tools. This is the first study which demonstrates a complete CRISPR/Cas9-based gene deletion from this bacterial genome. More importantly, a poultry experimental model was employed to assess the virulence potential of this mutant strain (ΔssaU_*S*G18) which was unable to produce any mortality in the experimentally challenged birds as compared to the wild type strain. No effect on weight gain was observed whereas bacteria were unable to colonize the intestine and liver in our challenge model. This in vivo loss of virulence in mutant strain provides an excellent functionality of this system to be useful in live vaccine development against this resistant and patho genic bacteria.

## 1. Introduction

*Salmonella enterica sub* sp. *enterica* serovar Gallinarum biovar Gallinarum (*S.* Gallinarum) is a crucial pathogen of poultry causing fowl typhoid (FT), an acute septicemic disease leading to catarrhal enteritis, hepatitis, pericarditis and splenomegaly [1]. With a mortality rate of 40–90%, the disease causes havoc in terms of decreased weight gain in broilers and low egg production in layer birds [2]. This disease spreads through both horizontal and vertical transmission [3]. Currently, live attenuated strain (9R) with a semi-rough lipo-polysaccharide structure has been used as vaccine to control FT in poultry [4,5]. In the last two decades, various outbreaks of FT have been reported in India, Nigeria, Korea and Japan [4,5,6,7]. In addition, the emerging resistance of *S*. Gallinarum against ciprofloxacin, amoxicillin, erythromycin, tetracycline and doxycycline reported in recent studies demonstrates an alarming situation in the future for poultry industry [8]. This urges the undermining of the virulence factors and molecular pathways that contribute to *S*G’s pathogenicity. The enormous 85 kb plasmid of *Salmonella* Gallinarum is crucial for virulence, and various plasmid-borne virulence genes have been characterized for their virulence potential [9]. Salmonella pathogenicity island 2 (SPI-2) has been shown to play a crucial role in disease transmission by *Salmonella* enterica via type III secretion system apparatus (TTSS) [10].

The SPI-2 encoded TTSS gets activated and interfere with the maturation of phagosome once the *Salmonella* is engulfed. This results in the formation of specified *Salmonella* Containing vacuole (SCV), leading to intracellular survival and replication of *Salmonella* [11].

A number of steps precede the SCV formation, including endocytic pathway hence inhibiting the delivery of hydrolytic enzymes; relocation of SCV to perinuclear area and development of Salmonella-induced-filaments (Garcia-del Portillo et al., 1993). Due to bacterial intervention with many cellular processes these events proceeds to the survival, colonization, and spread of *Salmonella*. Hence SPI-2-encoded TTSS is crucial to orchestrate SCV formation and intracellular survival of *Salmonella* [12].

The genes belonging to SPI-2 including *ssaG, ssaV, SsaU* and *ssrA* have been classified as potential virulent factors for *Salmonella* infections [13]. These TTSS-associated genes regulate the trafficking of several virulence-related effector proteins from bacteria to host cells [14]. Translocator proteins are involved in the trafficking of another set of proteins directly into the host cytoplasm through their needle like injectisome. After translocation, these “effector proteins” act as the virulence mediator affecting the changes in the host cells [15].

In this study, complete CRISPR/Cas9-based knockout ΔssaU_*S*G18 strain was employed for a poultry experimental infection model to investigate the effect of the SPI-2 TTSS on the virulence of S. Gallinarum infections. Here, we demonstrate that a functional SPI-2 TTSS is necessary for the development of fowl typhoid and that ΔssaU_*S*G18 is incapable of colonization and death in chickens.

The CRISPR system which was discovered as a bacterial immune system usually consists of the (CRISPR-associated) Cas protein (typically Cas9) (type IIA), an RNA-dependent endonuclease which cleaves the target DNA determined by the CRISPR RNA (crRNA) dictating the region of double stranded break (DSB) through its spacer sequence (20 nucleotide) and a trans-activating (tracrRNA) which forms a complex structure with crRNA to guide the Cas nuclease [16]. Nowadays this crRNA and tracrRNA can be fused together to transcribe a functional single chimeric guide RNA (sgRNA) [17], which form a complex with Cas9 protein directing it to specified DNA locus by base pairing with protospacer adjacent motif (PAM). PAM motif consists of a few base pairs with their specific sequence and position which vary with defined CRISPR/Cas9 system [18]. In the type II system of the *S*.pyogenes, PAM refers to a –NGG- consensus sequence comprising of two (G:C) base pairs which occur one base pair downstream of the spacer binding site, within the target DNA [16]. Hence after the recognition, this complex mediates a double stranded break (DSB), three base pairs upstream of the PAM sequence. Since, DSB is lethal, cells during their cycle of repair can be exploited to acquire specific modification via homologous recombination or integrated homology-directed repair (HDR) via a PCR product, plasmid or oligonucleotide [19].

Phage-derived lambda recombination system has emerged as a useful and alternate method for the chromosomal genome engineering [20,21]. The method relies upon the phage recombination proteins which utilize short homology region at 5′ end of linearized DNA fragment to commence the homology directed recombination. The use of a phage-derived recombination system has already been reported in many pathogenic organisms such as *E*.coli, *Salmonella* and *Yersinia* species [22,23,24]. More recently, the lambda red recombination based genome deletion was also reported in *Salomella* enterica serovar Pullorum in which the cigR deletion mutant was further tested for its virulence and biofilm formation [25]. The lambda red genes (i.e., gam, bet, and exo) from bacteriophage lambda are vital to support homologous recombination of linearized dsDNA [26]. Gam retards host endogenous nuclease activities of RecBCD and SbcCD; shielding the dsDNA for recombination apparently avoids the degradation of linear dsDNA template in the cell [27,28]. The Exo acts as an exonuclease which truncates linearized dsDNA in 5′→3′ direction from both ends, forming 3′ single-stranded tails for dsDNA [29]. Beta swiftly binds the ssDNA template ends to complementary strand to target DNA [30].

In this study, we have employed a simplified genome engineering strategy using CRISPR/Cas9 parallel with the conventional lambda red recombineering. This pairing increases the genome editing efficiency of the overall system with shorter time period required than previously reported methods [31]. An animal infection model was used to study the effect of *ssaU* mutant on the pathogenicity of this bacteria. This study demonstrates the versatility of CRISPR/Cas9 as a robust bioengineering tool for attenuated live vaccine development by targeting the different virulence-associated genes of pathogenic and resistant bacteria.

## 2. Materials and Methods

### 2.1. Strains, Culture Conditions, Plasmids, and Oligos

All the bacterial cultures along with the plasmids used in this study are mentioned in the Table 1. *E.* coli Top 10 cells used for transformation were cultured at 37 °C in Tryptic Soy Broth (TSB), supplemented with 100μg/mL of ampicillin or 50 μg/mL of kanamycin as required. *S.* Gallinarum (wild type) strain and transformants were grown aerobically at 37 °C in TSB supplemented with 50 μg/mL of kanamycin (kan), 100 μg/mL of ampicillin (amp) or chloramphenicol (cam) 25 μg/mL as per requirement. All the DNA oligonucleotide sequences used in this study are listed in Table 2. The linear DNA fragments designed for DSB repairing were amplified through Q5^®^ High-Fidelity DNA Polymerase (NEB) after fusion using NEBuilder^®^ HiFi DNA Assembly as described in the Supplementary Material.

### 2.2. DNA Editing Template Construction, gRNA Cloning and Induction Method

**Figure 1:**
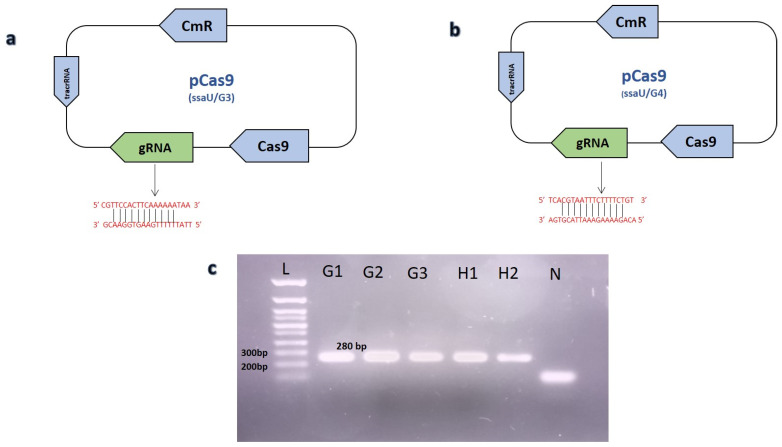
Confirmation of gRNA cloning in pCas9 through PCR. (**a**) Graphical representation of ssau/G3 plasmid showing spacer cloning using golden gate assembly. (**b**) The plasmid map of ssaU/G4 along with its spacer sequence. (**c**) PCR confirmation of gRNA cloning in pCas9. Lane (G1-G3) illustrating positive result with 280 bp amplified fragment, showing cloning of gRNA3 in ssau/G3 plasmid, while lane (H1-H2) represents the successful cloning of second gRNA named ssaU/G4 plasmid. Lane (N) represents negative control. Lane (L) shows 100 base pair plus DNA ladder (Bioron).

Homology arms ~1800 bp as DNA editing template were cloned in the pET22b (+) vector. Homology arms flanking up and downstream regions of the selected *ssaU* gene was amplified through overlap primers HA1_ssaU_Fp and HA1_ssaU_Rp for upstream while HA2_ssaU_Fp and HA2_ssaU_Rp for downstream region followed by fusion through NEB Hifi DNA^®^ assembly kit (see Supplementary Material). This assembly mixture was later amplified by using primer HA1_ssaU _Fp and HA2_ssaU_Rp and cloned in pET22b (+) vector named as RP-18. For dsDNA repair template, PCR product of fused homology arm fragments was used after PCR purification. In order to introduce targeted DSB, RNA spacer (5′-CGTTCCACTTCAAAAAATAA-3′) and (5′-TCACGTAATTTCTTTTCTGT-3′) were cloned in pCas9 and named as ssaU/G3, ssaU/G4, respectively. A Golden Gate assembly protocol was used for cloning of space sequence in plasmid (see Supplementary Material) [32,33]. As off target effects of gRNA has been reported, which could lead to secondary DSB or mutations, we used online available software Cas-OFFinder [34] (Bae et al., 2014) to rule out any secondary target site possibilities. Successful cloning of spacer sequence in pCas9 was confirmed by using primers Cas9_R as reverse and gRNA3F_ssaU or gRNA4F_ssaU as forward primers for ssaU/G3 and ssaU/G4, respectively (Figure 1). Spacer cloning was confirmed by Sanger sequencing (Supplementary Material Figure S1).

Mutants were identified using screening primers designed outside of the frame of homology arm sequence named ssaU _screening_Fp and ssaU _screening_Rp. Mutants were further confirmed by sanger sequencing.

In all of the experiments exhibiting recombineering, controls were kept in place to assess the electroporation of plasmids. At least three replicates were employed to assess the recombination of the plasmid/dsDNA template for each recombineering experiment performed. The formula for calculating transformation and electroporation efficiency is provided in the Supplementary Material. Statistical Analysis including arithmetic means with SD and *p*-value using two-way ANOVA was performed using the software tool Graphpad prism version 8.0.1 GraphPad Software, La Jolla, CA, USA, www.graphpad.com (accessed on 20 July 2022).

### 2.3. Mutant Strain Preparation

The red recombineering plasmid (pRed) was electroporated into the S. Gallinarum cells selected on 50 μg/mL kanamycin and confirmed through colony PCR using Pcas-red-Fp and Pcas-red-Rp as forward and reverse primers, respectively. Overnight cultures of the recombineering plasmid transformed S. Gallinarum cells (500 μL) were shifted to 30 mL TSB at 30 °C for ~1.5 h with shaking at 200 rpm. When OD_600 nm_ of cells reached 0.2, the cells were induced by 10 mM arabinose for expression of lambda recombineering components (Exo, Gam, Beta) and further incubated at 30 °C for ~1 h with shaking. These cells were then used for electrocompetent cells preparation.

### 2.4. Electroporation

A single colony of the wild type *S*. Gallinarum was inoculated in 3 mL TSB overnight. Overnight culture from this single colony was diluted (500 μL) in 50 mL of the TSB and incubated at 37 °C with shaking at 200 rpm. When OD_600_ of the cells reached 0.2–0.3, the cells were subjected to chilled ice for ~10 min and later centrifuged at 5000 rpm for 5 min at 4 °C. The supernatant was discarded and cells were washed with 20 mL sterile distilled water once and twice with sterile 10% glycerol solution at 4 °C. Finally, the cells were resuspended in 1 mL 10% glycerol solution and 100 uL aliquots were stored at −80 °C.

According to electroporation requirements, aliquots were thawed on ice for 10 min. Then the cells were mixed with 8 μg of the required plasmid/linearized DNA and transferred to pre-chilled 2 mm electroporation cuvette (Bio-Rad). Cells were then pulsed at 25 KV/cm, 1100 Ω, and 25 μF, with time constant of 5.0 milli-second. Immediately after the pulse, cells were mixed with the TSB (1 mL) and incubated at 37 °C for ~2 h, which were then plated on TSA plates containing corresponding antibiotics. Plates were incubated at 37 °C for 24–36 h or until the colonies were evident. Mutants were screened using cPCR.

### 2.5. Poultry Experimental Model to Evaluate Virulence

To infect broiler birds, wild type *S.* Gallinarum (WT-SG18) and modified (ΔssaU_SG18) were used. A total of 200 disease-free male broiler chickens of the Cobb 500 strain were used in this study which were procured from a commercial hatchery. The birds were kept in sterile pens in an environmentally-controlled room with a 20-h light cycle. The birds were distributed randomly into four groups: SG_WT (S. Gallinarum positive control), SG_Negative (not infected with any pathogens), complement strain (cSG18) and ΔssaU_SG18 mutant group, with five equal sized replicates containing 10 birds each on the first day. On day 7, cloacal swabs were used to randomly sample birds for the screening of *S*G infection. Throughout the duration of the experiment, the birds had unfettered access to antibiotic-free food and water. At 16 days of age, birds were administered 1 mL of normal saline containing approximately 1 × 10^8^ colony-forming units (CFU) of each strain via oral gavage. Birds were fed orally with *S*G in order to mimic the natural route of infection. After administration, birds were routinely observed for gross signs of disease. Throughout the trial, birds were weighed each week to record any changes in weight gain. Gross examination and post-mortem investigations of both dead and critically ill birds were conducted 21 days post-infection.

## 3. Results

### 3.1. Lethality of Cas9 Induced Double Stranded Break and Efficiency of Repair Plasmids

We employed multiple techniques for optimizing CRISPR/Cas9-mediated genome editing in the *S*G, by targeting the selected *ssaU* gene encoding type three secretion system apparatus (TTSS), which is located on *Salmonella* pathogenicity island 2 (SPI-2) comprising 1059 nucleotides. Initially, two plasmid approach was employed in which the modified pCas9 plasmid [35] containing the constitutively expressing Cas9 nuclease for DSB and repair template plasmid RP-18 was used.

Co-electroporation of the modified pCas9 ssaU/G3 and RP-18 (DNA editing plasmid) into SG cells resulted in no clones. However, colonies consistently appeared on the control plate when electroporated with simple pCas9 plasmid (with no guide RNA) and RP-18, with transformation efficiency of (8.2 × 10^2^ ± 25 cfu/μg *n* = 3), which rule out the toxic effect of empty Cas9 nuclease and suggest the lethality of Cas9 nuclease due to cloned gRNA causing genomic DSB [32]. Clones were not evident even after utilizing a higher amount (10 μg) of repair template plasmid or decreasing the ssaU/G3 (< 50 ng) concentration.

This suggested that the DSB induced by ssaU/G3 was not resolved by the homology-directed repair, meanwhile native non-homologous end joining (NHEJ) was also not efficient in the SG [36]. We further cloned a second spacer RNA in pCas9 (ssaU/G4) targeting another region of the *ssaU* gene (Figure 1), however, no colonies were observed after electroporation with the plasmid based homology templates RP-18. A two-step electroporation was also performed, where RP-18 was electroporated first into SG to allow the cross-over event to occur before the electroporation of ssaU/G3 or ssaU/G4. However, this strategy still yielded no clones.

### 3.2. Coupling CRISPR/Cas9 with Lambda Red Recombineering Results in Successful Gene Knock Out

**Figure 2:**
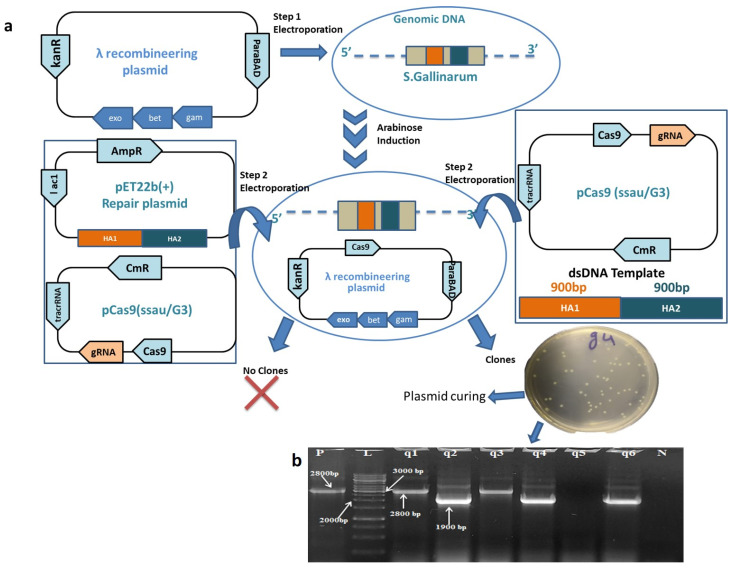
Coupling CRISPR/Cas9 machinery with lambda recombineering for efficient gene deletion from S. Gallinarum genome. (**a**) Stepwise strategy used for deletion of ssaU gene from SG genome using lambda recombineering and CRISPR/Cas9 system. Step (1) indicating the electroporation of pRed. Step (2) co-electroporation of (ssaU/G3) along with RP-18 DNA editing plasmid in SG does not yield any edited clones while co-electroporation of ssaU/G3 combined with dsDNA editing template ≃1800 bp yielded > 50 clones. (**b**) Colony PCR of clones obtained after co electroporation of ssaU/G3 along with double stranded DNA editing template. Lane (q2, q4, q6) indicated gene deletion with a band length of 1.9 kb. While lane (q1, q3) showed no gene deletion with fragment length of ≃2.8 kb. Lane (N) shows negative control reaction. Lane (P) indicate positive control reaction using SG wild type genome as DNA template amplifying a product of ≃2.8 kb. Lane (L) indicates a 1 Kb DNA ladder (Thermo fisher Scientific).

To overcome the lower recombination capacity of the bacterial cells after DSB, we used the lambda red recombineering plasmid pRed available in our lab amplified from pCas in combination with pCas9 [37]. After the induction with 10 Mm arabinose and followed by the co-electroporation with ssaU/G3 (300 ng) and linearized dsDNA repair template (8 μg) clones were apparent on the selection plate. The colonies that appeared were screened through colony PCR. Surprisingly a maximum of (70 ± 2.5% *n* = 3) clones showed successful deletion of the said gene. The PCR screens of the *ssaU* gene deletion is shown in (Figure 2), unraveling the high potential of this unified system for efficient gene deletion in *S*. *Gallinarum.*

Deletion of the *ssaU* gene was further confirmed through Sanger sequencing (Supplementary Material Figure S2), whereas no mutation was observed in the flanking upstream and downstream regions of the deleted gene confirming the precise genome modification.

### 3.3. Clearance of ssaU Mutant Strain from Experimental Birds

**Figure 3:**
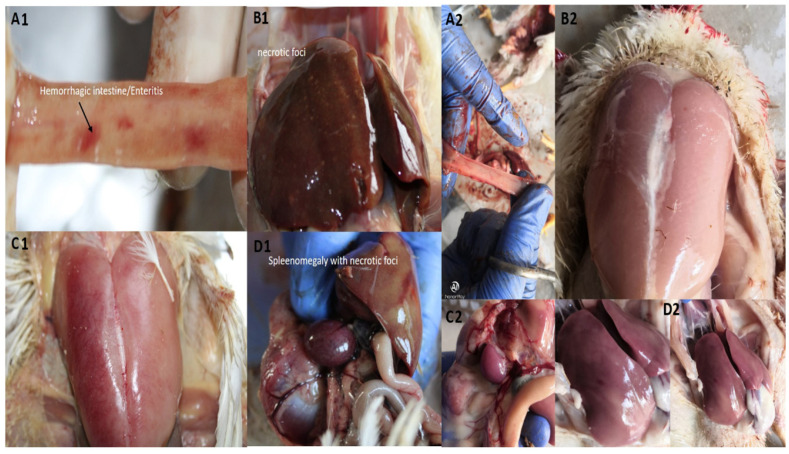
Gross pathological examination of dead experimental birds. Birds infected with WT-SG18 showed (**A1**) intestinal hemorrhages, (**B1**) necrotic foci on liver with discoloration, (**C1**) fever and inflammation (**D1**) splenomegaly. In contrast Δssau_SG18 infected birds (**A2**,**B2**,**C2**,**D2**) showed no gross pathological lesions of intestine, muscles, spleen and liver, respectively.

In order to determine the systemic infection, bacteria were isolated from visceral organs of the birds, primarily the liver and ileum, after 4, 7 and 14 days post-infection. The WT-SG18 strain recovered from the liver and intestine of infected birds (positive control group) showed 1.3 × 10^3^, 5.0 × 10^5^ and 1.6 × 10^8^ CFU/g after 4, 7 and 14 days, respectively, post-infection indicating systemic bacteremia. Our results are in agreement with the previously reported study showing the highest number of viable counts that were isolated from the liver after 14 days post-infection with *S*. Gallinarum [38]. In contrary, ΔssaU_SG18 strain could not be recovered from any of the tissues, even following enrichment with selenite broth, although it was recovered from the caecum 2 days post-infection. The post-mortem examination of WT-SG18 group exhibited the typical signs of fowl typhoid pathology (Figure 3), including hepatosplenomegaly with necrotic foci on liver and spleen, bronze discoloration of liver and hemorrhages in the anterior small intestine. In contrast, the ΔssaU_SG18 strain showed no signs and symptoms of disease at postmortem examination throughout the period of experiment (Figure 3).

**Figure 4:**
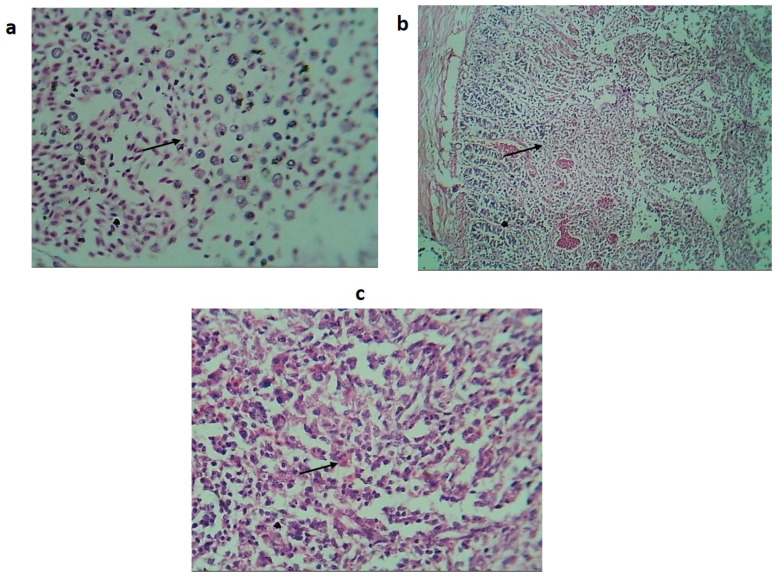
Histopathological findings of birds orally treated with WT-SG18. (**a**) Histological section of the liver showing hemorrhage, vacuolar degeneration of hepatocytes (black arrow) and Infiltration of mononuclear inflammatory cells. (**b**) Histological section of intestine showing coagulative necrosis (black arrow). (**c**) Section of spleen showing hemorrhage, infiltration of mononuclear inflammatory cells and necrosis (black arrow) (H and E stain).

Histopathological analysis of the liver, spleen and intestine revealed lesions showing marked necrosis, hemorrhages, congestion and infiltration of heterophils (avian polymorphonuclear cells) characteristic of fowl typhoid (Figure 4) [39].

**Figure 5:**
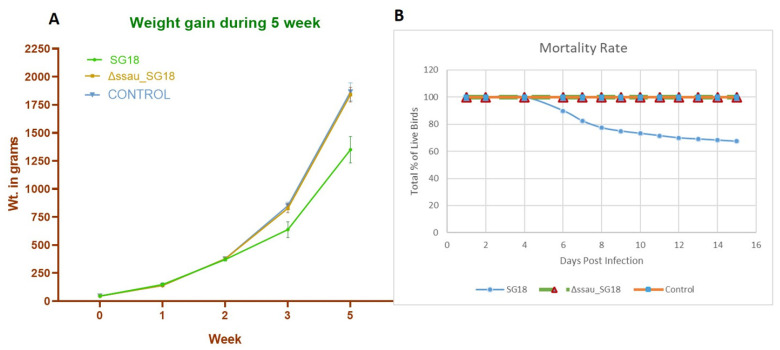
(**A**) Comparison of weight gain between WT-SG18, Δssau_SG18 mutant and control group at 5th week of experiment. (**B**) Progression of mortality in chickens recorded over 15 days post-infection with SG18 and ΔssaU_SG18 strains.

Our results suggest that the avirulent strain ΔssaU_SG18 was unable to invade from the intestine to the liver, which is consistent with a previously reported work where the avirulent *ssaU* mutant of *S*. Gallinarum was not recovered from tissues of birds, hence suggesting their rapid clearance after infection [40]. The particular loss of SPI-2 function in serovar Typhimurium leading to attenuation of typhoidal disease in mice models has been previously reported [41]. The complement strain (cSG18) which was constructed using pTZ19R to achieve continuous expression of *ssaU* showed virulence identical to that of WT-SG18, further confirming the role of this gene in the pathogenicity of bacteria. Weight gain and progression of mortality for both WT-SG18, negative control and ΔssaU_SG18 during the period of experiment is shown in (Figure 5). The final weight gain of ΔssaU_SG18 and negative control revealed no significant difference (*p*-value = 0.8166), whereas the WT-SG18 group exhibited higher significance (*p*-value < 0.0001) due to disease progression and systemic infection leading to abrupt loss in weight gain. The overall mortality data also showed higher significance (*p*-value < 0.0001), where no mortality was observed in the ΔssaU_SG18 group, while the WT-SG18 group demonstrated an overall mortality of 32 ± 4.4% starting at day 6 (Figure 5B).

## 4. Discussion

For the past 20 years, genetic engineering using recombineering has been employed as a robust tool to modify the bacterial genome [42]. More recently, the CRISPR/Cas9 system has highlighted its potential also as a powerful tool for the genetic manipulations and hence revolutionizing genome engineering. Therefore, we have demonstrated the use of such a coupled system to modify the genome of *S*G which provides a streamlined approach for knock-out mutant generation. The first approach to genetically manipulating the *S*G genome using two plasmids (ssaU/G3 and RP18) did not produce any mutant clones. We hypothesized that the plasmid-based DNA editing template was less efficient. The lower efficiency of plasmid-based repair templates has already been reported in the previous studies [43].

Considering this, we moved on to the lambda red recombineering proteins which have been proven to be influential in enhancing the recombination capacity of bacteria [44,45]. By using the *S*G cells expressing lambda protein along with ssaU/G3 and dsDNA template. The recombination efficiency in case of gene knockout was 70 ± 2.5% using the CRISPR/Cas9 and recombineering system together. We were unable to obtain any clone using only lambda recombination alone and not CRISPR/Cas9 system. The plasmid-based homologous recombination using CRISPR/Cas9 causes the vector integration event (VIE) problems (homologous recombination by double crossover), which further needs screening steps for the selection of the required positive mutant [17,32]. Additionally, the lambda red recombination alone depended upon the integration of the resistant marker, hence this coupled methodology provides a straightforward design for *S.* Gallinarum genome editing that too with higher efficiency. As this approach does not require construction of HDR plasmid, it provides a reliable methodology for precise knock-out production.

A complete deletion of the *ssaU* gene from *S*. Gallinarum resulted in in vivo attenuation; however, further deletion of other virulent genes from the Δssau_SG18 strain will make this strain a suitable candidate for vaccine design. TTSS is required for triggering apoptosis in the epithelial cells [46]. In both apoptosis (enteric phase) and pyroptosis (delayed macrophage death), TTSS is instrumental in the translocation of effectors into the host cell during the early phase of pathogenesis [47], whereas the effector proteins (*SpvB, SseL, SlrP*), which are crucial for host cellular dysfunction and deterioration, are expressed in the later stages of infections [48,49]. Several TTSS components have been found to be crucial for *SpvB*-induced actin-depolymerization and cellular cytotoxicity, lending support to the fact that *SpvB* is translocated into the host cell via TTSS [50,51]. The role of *SpvB* as potential virulent effector protein has previously been identified in our lab where *SpvB* mutant of *S*G was also unable to produce disease symptoms in the poultry infection model [52]. Hence, it can be speculated that the TTSS act as a vital machinery to deliver the effector proteins inside the host cell which give rise to the induction of virulence. Here we have highlighted the significance of the SPI-2 TTSS in a naturally occurring route of systemic, typhoid-like Salmonella infection that has economical and veterinary significance in developing countries. Following this optimized CRISPR/Cas9-based manipulation, studies are underway in our lab to produce an indigenous vaccine strain by deletion of a larger pathogenicity island to control this disease which has been a major concern for Pakistan poultry industry.

## 5. Conclusions

This study shows the importance of the SPI-2 TTSS in the infection of typhoid-like salmonellosis that has significant economic and veterinary importance in under developed countries. The SPI-2 TTSS allows the serovar Gallinarum to cause the disease by facilitating the bacterial survival within the macrophages and reticuloendothelial system. This coupled CRISPR/Cas9 and lambda recombineering system can be formulated for the live vaccine development against indigenous *S.* Gallinarum by large-scale deletion of *Salmonella* pathogenicity islands or relative virulence genes [32].

## Figures and Tables

**Table 1:** Plasmids and bacterial strains used in this study.

Sr. No.	Strain/Plasmid	Characteristics	Reference/Source
1.	*E*.coli Top 10	Tetracycline, hsdR, mcrA	Thermofisher Scientific
2.	*Salmonella* Gallinarum	Poultry origin	Addgene
3.	pCas9	pACYC184, Chloramphenicol, DH5alpha	Addgene (Plasmid #42876)
4.	pRed	Kanamycin, Lambda Red recombinase, DH5alpha, RepA101ts	This study
5.	pET22b (+)	pelB, His•Tag^®^, Ampicillin	Addgene (Plasmid # 69744-3)
6.	RP-18	pelB, His•Tag^®^, Ampicillin, 1.8 kb HAs	This study
7.	ssaU/G3	pACYC184 Chloramphenicol, gRNA3	This study
8.	ssaU/G4	Chloramphenicol, pACYC184, gRNA4	This study

**Table 2:** Oligonucleotide sequences designed for this study.

Oligonucleotide	Sequence (5′–3′)
HA1_ssaU_FP	aaaatcTCTAGAAGCGGTATCCTGTTGAATTATACC
HA1_ssaU_Rp	AATAACGTTTCAGGAATTTTATCTCTTCttttctgtagtctgttctgttttc
HA2_ssaU_Fp	gaaaacagaacagactacagaaaaGAAGAGATAAAATTCCTGAAACGTTATT
HA2_ssaU_Rp:	ttatgaCTCGAGTGCTGCTTGCTGCGGTTTACCAGA
gRNA3F_ ssaU	aaacTTATTTTTTGAAGTGGAACGg
gRNA3R_ ssaU	aaaacCGTTCCACTTCAAAAAATAA
gRNA-4F-ssaU	aaacACAGAAAAGAAATTACGTGAg
gRNA-4R- ssaU	aaaacTCACGTAATTTCTTTTCTGT
ssaU _screening_Fp:	ACGTCTATGCCGGTAGTGTTGGT
ssaU _screening_Rp:	CATTTGTATGGCTGTGGTTACCG
Cas9_R	ATAGTGACTGGCGATGCTGTC
Pcas-red-Fp	ATTCACTTTTTCTTCACAACCG
Pcas-red-Rp	TATCACCAGTGGGTTTACTTTC
lambda-Fp	AAGCAGACAGGACATGAGCGGATACATATTTG
lambda-Rp	CGCTCATGTCCTGTCTGCTTACATAAACAG
ssau_seq-Fp	GTATCAGCTTCTCTCTTCCT
ssau-seq-Rp	CACCTTTATCGTCAAGCACT

(Underline indicates restriction recognition sequence; lowercase indicates overhang regions).

## Data Availability

Further details related to primer designing and plasmid used, including additional photographs of experiments are provided.

## Supplementary Materials

The following supporting information can be downloaded at: https://www.mdpi.com/article/10.3390/biomedicines10123028/s1, Figure S1: Confirmation of gRNA cloning by Sanger sequencing., Figure S2: Confirmation of precise gene knock-out by Sanger sequencing, Calculation of transformation and recombination efficiency, Detailed protocol for Genome engineering of *Salmonella* Gallinarum.

## Abbreviations

CRISPR, Clustered Regularly Interspaced Short Palindromic Repeats; Cas, CRISPR-associated; cPCR, colony PCR; DSBs, double-strand breakages NHEJ, non-homologous end-joining; ORF, open reading frame; PAM, protospacer adjacent motif; vector integration event; WT, wild type; PAM, protospacer adjacent motif; SP1-2, Salmonella pathogenicity island 2; TTSS, Type Three Secretion System.
