# Supplementary material for: Coupling CRISPR/Cas9 and Lambda Red Recombineering System for Genome Editing of Salmonella Gallinarum and the Effect of ssaU Knock-Out Mutant on the Virulence of Bacteria
Source: Biomedicines. 2022 Nov 24;10(12):3028. doi: 10.3390/biomedicines10123028 (PMC9776377; doi:10.3390/biomedicines10123028)

Transformation and recombination efficiency were calculated by using the following formula:

TE (%) (per/ug of plasmid) = No. of transformants / ug of DNA used * final volume at recovery (mL) / Volume plated (mL)

Recombination efficiency (%) = No. of clones showing positive mutation / Total clones screened × 100

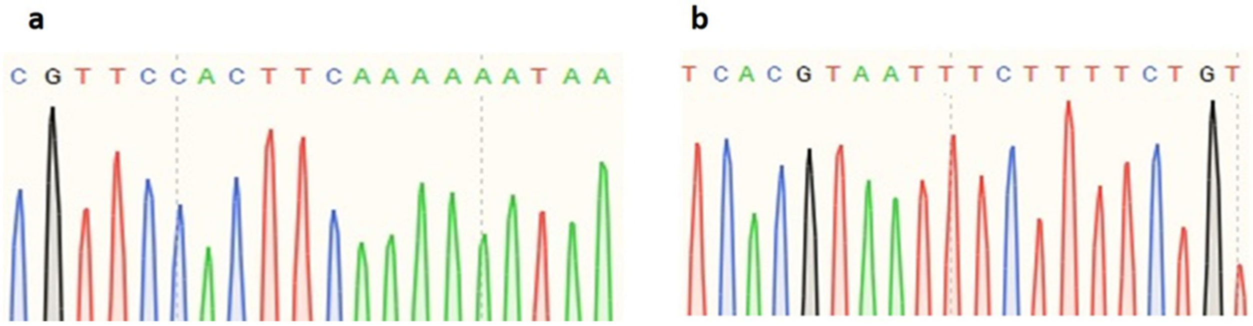

Figure S1. Confirmation of gRNA cloning by Sanger sequencing (**a**) Confirmation of ssau/G3 spacer cloning using Sanger sequencing. (**b**) Confirmation of ssau/G4 spacer cloning using Sanger sequencing.

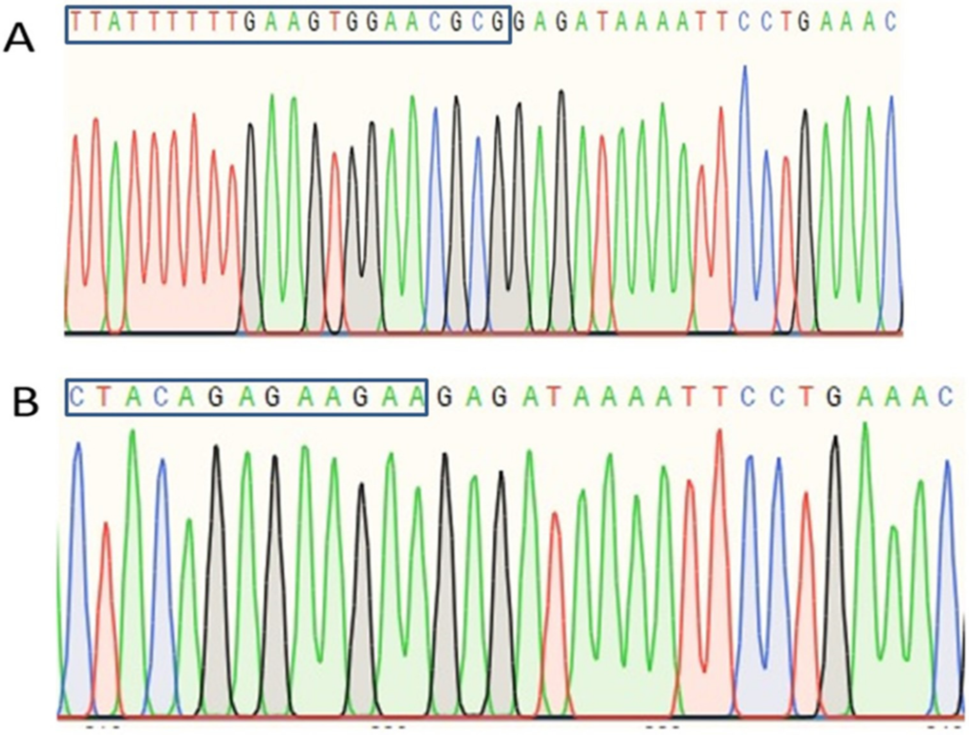

Figure S2. Confirmation of precise gene knock-out by Sanger sequencing. Changes in sequences indicated (**A**) Mutant strain (ΔssaU_SG18) While (**B**) indicate wild type SG18 (WT-SG18).

Detailed protocol for Genome engineering of *Salmonella* Gallinarum:

1. Amplification and Cloning of Homology Arms From SG Genome Into Respective Vector

Amplify the upstream and downstream of the target gene using *Salmonella* Gallinarum genomic DNA as template. Select ~500bp to 1 kb DNA sequence of the upstream and downstream of the target gene, respectively. Design the primers as described:

***ssaU*- Gene Cassette with Homology arms**.

TTTATGGATCGTCCTTTTTACGTCTATGCCGGTAGTGTTGGTGGCATCGGTAGTTGGTGTCATCGTAAGC
CTTGTTCAGGCCTTGACTCAAATACAGGACCAAACGCTACAGTTCATGATTAAATTATTGGCAATTGCAA
TAACCTTAATGGTCAGCTACCCATGGCTTAGCGGTATCCTGTTGAATTATACCCGGCAGATAATGTTACG
AATTGGAGAGCATGGTTGAATGGCACAACAGGTAAATGAGTGGCTTATTGCATTGGCTGTGGCTTTTATTCGA
CCATTGAGCCTTTCTTTATTACTTCCCTTATTAAAAAGTGGCAGTTTAGGGGCCGCACTTTTACGTA
ATGGCGTGCTTATGTCACTTACCTTTCCGATATTACCAATCATTTACCAGCAGAAGATTATGATGCATAT
TGGTAAAGATTACAGTTGGTTAGGGTTAGTCACTGGAGAGGTGATTATTGGTTTTTCAATTGGGTTTTGT
GCGGCGGTTCCCTTTTGGGCCGTTGATATGGCGGGGTTTCTGCTTGATACTTTACGTGGCGCGACAATGGGTAC
GATATTCAATTCTACAATAGAAGCTGAAACCTCACTTTTTGGCTTGCTTTTCAGCCAGTTCTTGTG
TGTTATTTTCTTTATAAGCGGCGGCATGGAGTTTATATTAAACATTCTGTATGAGTCATATCAATATTTA
CCACCAGGGCGTACTTTATTATTTGACCAGCAATTTTTAAAATATATCCAGGCAGAGTGGAGAACGCTTT
ATCAATTATGTATCAGCTTCTCTCTTCCTGCCATAATATGTATGGTATTAGCCGATCTGGCTTTAGGTCT
TTTAAATCGGTCGGCACAACAATTGAATGTGTTTTTCTTCTCAATGCCGCTCAAAAGTATATTGGTTCTA
CTGACGCTCCTGATCTCATTCCCTTATGCTCTTCATCACTATTTGGTTGAAAGCGATAAATTTTATATTT
ATCTAAAAGACTGGTTTCCATCTGTATGAGCGAGAAAACAGAACAGCCTACAGAAAAGAAATTACGTGATG
GCCGTAAGGAAGGGCAGGTTGTCAAAAGTATTGAAATAACATCATTATTTCAGCTGATTGCGCTTTATTTGTA
TTTTCATTTCTTTACTGAAAAGATGATTTTGATACTGATTGAGTCAATAACTTTCACATTACAATT
AGTAAATAAACCATTTTCTTATGCATTAACGCAATTGAGTCATGCTTTAATAGAGTCACTGACTTCTGCA
CTGCTGTTTCTGGGCGCTGGGGTAATAGTTGCTACTGTGGGTAGCGTGTTTCTTCAGGTGGGGGTGGTTATTGC
CAGCAAGGCCATTGGTTTTAAAAGCGAGCATATAAATCCGGTAAGTAATTTTAAGCAGATATTCTCTTTACAT
AGCGTAGTAGAATTATGTAAATCCAGCCTAAAAGTTATCATGCTATCTCTTATCTTTGCCTTT
TTCTTTTATTATTATGCCAGTACTTTTCGGGCGCTACCGTACTGTGGGTTAGCCTGTGGCGTGCTTGTGG
TTTCTTCTTTAATAAAATGGTTATGGGTAGGGGTGATGGTTTTTTATATCGTCGTTGGCATACTGGACTA
TTCTTTTCAATATTATAAGATTAGAAAAGATCTAAAAATGAGTAAAGATGACGTAAAACAGGAGCATAAAGA
TCTGGAGGGCGACCCTCAAATGAAGACGCGGCGTCGGGAAATGCAGAGTGAAATACAAAGTGGGAGTTTAG
CTCAATCTGTTAAACAATCTGTTGCGGTAGTGCGTAATCCAACGCATATTGCGGTTTGTCTTGGCTATCATCCC
ACCGATATGCCAATACCACGCGTCCTGGAAAAAGGCAGTGATGCTCAAGCTAACTATATTGTTAACATCGCT
GAACGCAACTGCATCCCCGTTGTTGAAAATGTTGAGCTGGCCCGCTCATTATTTTTTGAAGTGGAACGCGGAG
ATAAAATTCCTGAAACGTTATTTGAACCCGTTGCAGCCTTGTTACGTATGGTGATGAAGATAGATTATGCGCA
TTCTACCGAAACACCATAAATGCTTTTGGTATGCTTCTTCAGGCCACTGCGAAGGTTAAGAGGGTAATAGCGT
ATAGAGCAGTGCTTGACGATAAAGGTGAGAGACTGAAAATAATCGCTTTTAGCCTGGCACAAGCACCAGAT
AGCGTATTATAAAATTAAACAAGATAATGGATTGGTGCGTCTGAATGGACTCGAACCATCGACCCCCACCAT
GTCAAGGTGGTGCTCTAACCAACTGAGCTACAGACGCATTAAAATGATGGTGCGTTCAATTGGACTCGAACC
AACGACCCCCACCATGTCAAGGTGGTGCTCTAACCAACTGAGCTATGAACGCAACGTTGTAGGTGACAACGG
GGACGAATATTAGCGTCACAACCGCAATGAGGCAAGAGGGAAATCGCAATTTTCTTCCTGAAATCACCTGAT
TGCGGTGGAAATATGCAACATGTCGAGAAAATAGCCGCCATGCGACGGCTATCGTCGTATTATCGCGCAGCG

CGCTGCAAAATGATGGCGGACGGCTGACGTTGTAGATAGCGCATCCGTAGCATCATTAACACCGCCGCCGAG
GTCAGGCCGATGATGAACCCCATCCAGAAGCCTGCCGGTCCCATACGATCCACCACCAAATCCGTTAACGCC
AGGATATAACCGCTGGGTAAACCTAACACCCAGTAGGCGGTAAAGGTGATAAAAAAGATGGAACGCGTATC
TTTATAACCGCGCAGAATACCGCTGCCGATAACCTGTATAGAGTCGGAAATCTGGTAAACCGCAGCGAGCAG
CATTAATTGCGCGGCAAGCGCCACGACCTCAGGGTTGTCATTGTAGAGCAAAGCAATATGCTTACGCAGAGT
AACGGTAAAAATAGCGGTAACCACAGCCATACAAATGCCGACGCCTAAACCGGTACGCGCTGCGGTTTGCG
CATCCAGCGTTGAGCCCTGGCCCAGACGATAACCCACTCGAATCGTTACCGCCGCAGCCAGCGACATCGGCA
GTACGAACATCAG

(* Sequence in red indicate gene sequence, while black represents Homology arm sequence)

Right Homology Arm:

Fp: 5′-AAAATCTCTAGAAGCGGTATCCTGTTGAATTATACC-3′

Rp: 5′-CGATGGTTCGAGTCCATTCAGACGaattgttgtgccgaccgatttaaa-3′

Forward primer of HA1 contains restriction site for Xba1 along with additional bases (TCTAGA).

Reverse primer of HA1 contains overlapping sequence ~25bp for forward primer of HA2.

Left Homology Arm:

Fp: 5′-tttaaatcggtcggcacaacaattCGTCTGAATGGACTCGAACCATCG-3′

Rp: 5′-TTATGACTCGAGTGCTGCTTGCTGCGGTTTACCAGA-3′

Forward primer of HA2 contains overlapping sequence ~25 bp for reverse primer of HA1.

Reverse primer of HA2 contains restriction site for Xho1 along with additional bases (CTCGAG).

After amplification of targeted upstream and downstream regions purify the PCR products with the Thermoscientific gel purification kit (USA).

1.1 Hifi Assembly

Sr. No.	Component	Volume
1.	2X Hifi assembly reaction mixture	10 µl
2.	0.3 pmol HA1 (upstream of the target gene)	xx µl
3.	0.3 pmol HA2 (downstream of the target gene)	xx µl
4.	ddH2O to 20 µl	xx µl
	Total Volume	20 µl

Incubate at 50 °C for 1 hour in thermocycler.

After this 2 µL of this Hifi assembly reaction is used as a template for the amplification of assembled product using forward primer of HA1 (upstream) and reverse primer of HA2 (downstream).

For linearized dsDNA donor template PCR product was directly used after purification. Construction of the plasmid was verified by PCR or sequencing.

2. Cloning of Guide RNA in CRISPR Plasmids

All the CRISPR plasmids for gene deletion, single-nucleotide substitution and gene integration were assembled using the protocol described below.

2.1. Oligo Design

Select a 20 bp-spacer sequence before NGG (NGG is not included in the spacer) in the target gene. For this study we have used (https://chopchop.cbu.uib.no/) software to obtain the spacer sequence for required gene. Use the fasta sequence in the software. gRNA sequence without the -NGG sequence is selected. As the pCas9 plasmid has the Bsa1 cloning site to clone the spacer sequence, synthesize the two oligos in the following form.

Oligo design for cloning spacer sequence in pCas9 plasmid

5′- AAACNNNNNNNNNNNNNNNNNNNNG -3′
3′-NNNNNNNNNNNNNNNNNNNNCAAAA -5′

2.2. Phosphorylation

Sr. No.	Component	Volume
1.	oligo I (50 µM)	2 µL
2.	oligo II (50 µM)	2 µL
3.	10x T4 DNA ligase buffer (NEB)	5 µL
4.	T4 polynucleotide kinase	1 µL
5.	ddH2O	40 µL
	Total Volume	30 50 µL

Incubate the above reaction at 37ºC for 1 hour in thermocycler.

2.1 Annealing

Add 2.5 µl of 1 M NaCl to the phosphorylated oligo pairs.
Incubate the reaction tubes at 95 ºC for 3 min and slowly cool down to room temperature.
(Or, after the completion of 3 min take out the reaction tubes and let it cool down slowly at the room temperature.)
Dilute the annealed oligos 10 times using ddH2O.

Sr.no.	Component	Volume
1.	20 fmol CRISPR plasmid	xx µL
2.	Diluted annealed oligos (100 fmol)	1 µL
3.	10x T4 DNA ligase buffer (NEB)	1 µL
4.	T4 DNA ligase (NEB)	0.5 µL
5.	BsaI-HF (NEB)	0.5 µL
6.	dH2O to 10 µL	xx µL
	Total Volume	30 50 µL

2.4 Golden Gate Assembly Reaction (Cloning in pCas9)

After properly mixing the reagents run the following setup on thermocycler.

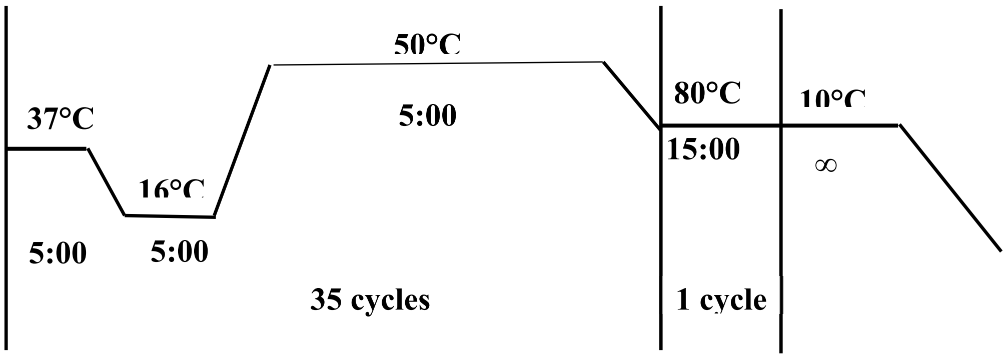

2.5. Transformation into Top 10 E. coli Cells

10 μl reaction product of Golden Gate assembly was transformed into 100 μl Top 10 E.coli competent cells. The successful colonies were selected on a TSA plate containing chloramphenicol 25μg/ml for pCas9. Colonies for gRNA cloned plasmid was verified by PCR and subsequent sequencing.

2.6. Plasmid Curing

A colony of the confirmed mutant that contains the pCas9 plasmid was incubated in TSB at 37 °C overnight. The following day, 5 μl culture was diluted in 5 ml TSB, and incubated at 42 °C until the culture was evident for growth. Therein the culture was later streaked onto a TSB agar plate and incubated at 37 °C overnight. The curing of the pCas9 plasmid was confirmed by streaking the colonies on TSB agar plates in the presence or absence of chloramphenicol (25 μg/ml). Cells were grown at 42°C in replicates until no growth was observed on chloramphenicol replica plates.

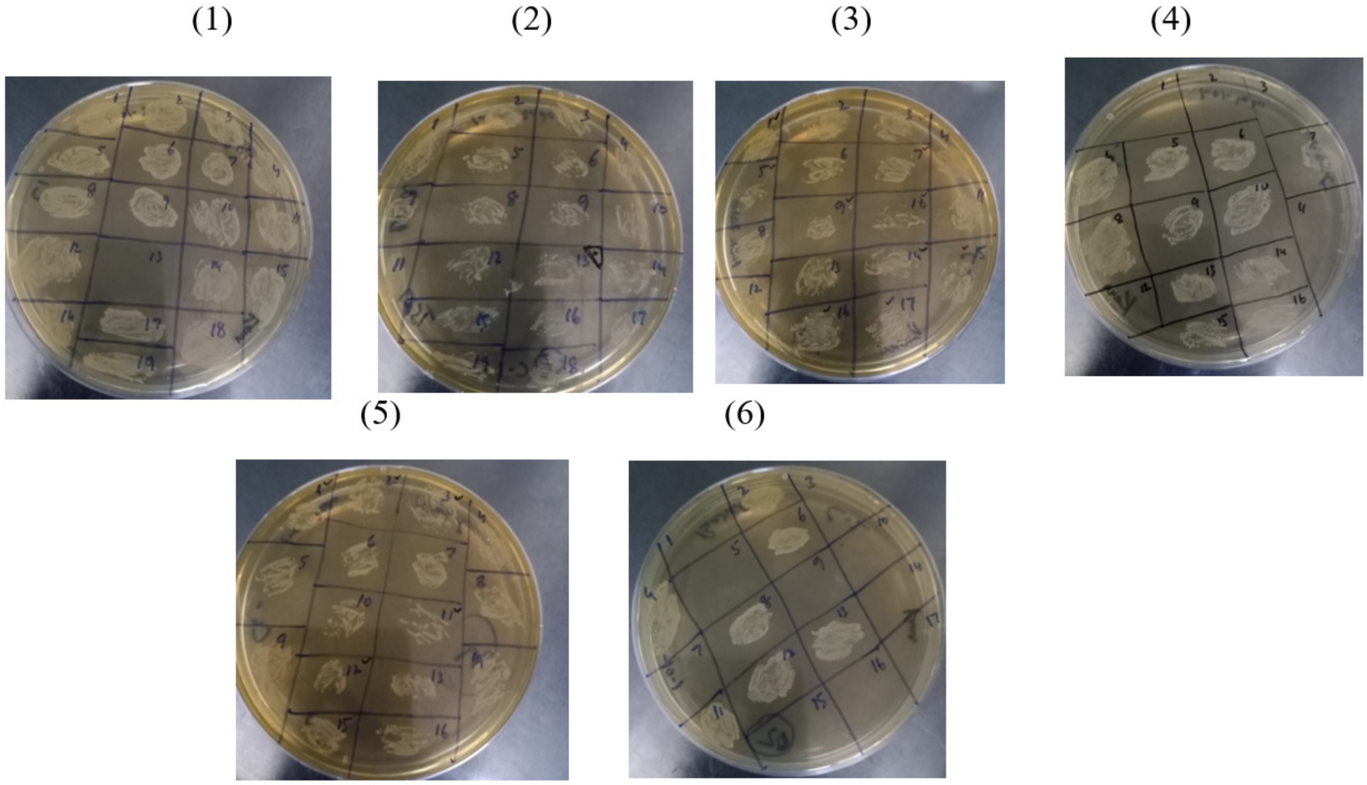

Supplement: Supplementary file 1 [file biomedicines-10-03028-s001.zip › biomedicines-2035681-supplementary.pdf]
